# What part of the total care consumed by type 2 diabetes patients is directly related to diabetes? Implications for disease management programs

**DOI:** 10.5334/ijic.675

**Published:** 2011-12-14

**Authors:** Christel E. van Dijk, Robert A. Verheij, Ilse C.S. Swinkels, Mieke Rijken, François G. Schellevis, Peter P. Groenewegen, Dinny H. de Bakker

**Affiliations:** NIVEL, Netherlands Institute for Health Services Research, Utrecht, The Netherlands; NIVEL, Netherlands Institute for Health Services Research, Utrecht, The Netherlands; NIVEL, Netherlands Institute for Health Services Research, Utrecht, The Netherlands; NIVEL, Netherlands Institute for Health Services Research, Utrecht, The Netherlands; NIVEL, Netherlands Institute for Health Services Research, Utrecht, The Netherlands and Department of General Practice, EMGO Institute for Health and Care Research, VU University Medical Center, Amsterdam, The Netherlands; NIVEL, Netherlands Institute for Health Services Research, Utrecht, Department of Sociology, Department of Human Geography, The Netherlands and Utrecht University, Utrecht, The Netherlands; NIVEL, Netherlands Institute for Health Services Research, Utrecht, The Netherlands and Tilburg University, Scientific Centre for Transformation in Care and Welfare (TRANZO), Tilburg, The Netherlands

**Keywords:** diabetes, disease management program, healthcare standards

## Abstract

**Background:**

Disease management programs (DMP) aim at improving coordination and quality of care and reducing healthcare costs for specific chronic diseases. This paper investigates to what extent total healthcare utilization of type 2 diabetes patients is actually related to diabetes and its implications for diabetes management programs.

**Research design and methods:**

Healthcare utilization for diabetes patients was analyzed using 2008 self-reported data (n=316) and data from electronic medical records (EMR) (n=9023), and divided whether or not care was described in the Dutch type 2 diabetes multidisciplinary healthcare standard.

**Results:**

On average 4.3 different disciplines of healthcare providers were involved in the care for diabetes patients. Ninety-six percent contacted a GP-practice and 63% an ophthalmologist, 24% an internist, 32% a physiotherapist and 23% a dietician. Diabetes patients had on average 9.3 contacts with GP-practice of which 53% were included in the healthcare standard. Only a limited part of total healthcare utilization of diabetes patients was included in the healthcare standard and therefore theoretically included in DMPs.

**Conclusion:**

Organizing the care for diabetics in a DMP might harm the coordination and quality of all healthcare for diabetics. DMPs should be integrated in the overall organization of care.

## Introduction

The number of people with chronic diseases, including diabetes mellitus, is increasing worldwide due to aging and an increasing number of people with overweight and physical inactivity. In 2030, the worldwide percentage of diabetes patients is estimated to be 4.4%, which would be an increase of 60% in comparison with the year 2000 [1]. Due to the high burden of chronic diseases for healthcare costs and impact on quality of life, management of chronic diseases has been an important issue in health policy in many countries [2]. While in the US almost all commercial health plans and large employers offer some form of disease management program (DMP) [3], public purchasers of health services have only recently implemented or are planning to implement DMPs [4, 5].

According to the Disease Management Association of America (DMAA) disease management is defined as a system of coordinated healthcare interventions and communications for populations with conditions in which patients self-care efforts are significant. Disease management supports the physician or practitioner/patient relationship and plan of care, emphasizes prevention of exacerbations and complications through the use of evidence-based practice healthcare standards and patient empowerment, and evaluates clinical, humanistic, and economic outcomes on an ongoing basis with the goal of improving overall health [6]. DMPs are expected to be the solution for the inadequate coordination of care between health services, variation in quality of care and increasing costs for chronic illnesses [4].

In the Netherlands, disease management was promoted with the nationwide introduction of disease oriented funding of chronic care in January 2010 (experimental since 2007), also known as bundled payments. Lack of collaboration between different healthcare providers partly explained by the fragmented organization of chronic care and funding urged the need for a new system of payment and organization for chronically ill patients. The basic idea behind disease oriented funding is that care for specific diseases can be organized around the needs of patients by stimulating the multidisciplinary coordination of care between healthcare providers within primary and between primary and secondary care. Within disease oriented funding, care is provided according to the national multidisciplinary evidence-based healthcare standard agreed between healthcare providers and patient organizations. Healthcare standards provide information about prevention and treatment of a specific health condition based on scientific evidence. To assess the quality of provided care, feedback and benchmarking reports have to be provided. In this way, disease oriented funding stimulates the further implementation of disease management. Care has to be organized by a group of healthcare providers (who form a legal entity) who negotiate a lumpsum remuneration per patient with insurers. Groups of healthcare providers can provide care themselves or subcontract other healthcare providers [7, 8]. Theoretically, these healthcare providers do not have to be the patient’s personal GP, although in the Netherlands all inhabitants are obligatory listed with a GP-practice. Disease oriented funding was introduced for type 2 diabetes and cardiovascular risk management in January 2010 and for COPD in July 2010. This article focuses on diabetes management.

Since many type 2 diabetes patients also suffer from other chronic and acute illnesses [9, 10], only part of the healthcare for these patients will probably be included in disease oriented funding and in the disease management program. Organizing or financing only a part of healthcare for a patient could affect the quality and coordination of care on patient level, and thereby healthcare outcomes. The objective of this article is to examine to what extent total healthcare utilization of type 2 diabetes patients is actually related to diabetes and therefore theoretically part of a diabetes management program in disease oriented funding and to discuss its implications for DMPs in general.

## Methods

### Study design

For the purpose of this study, healthcare utilization of known type 2 diabetes patients in the Netherlands was assessed in 2008 and compared to the Dutch type 2 diabetes multidisciplinary healthcare standard to establish which part of this care would be included in a DMP for diabetes. This study took place before the official introduction of disease oriented funding. In the Netherlands, no national database exists that provides information about all components of healthcare utilization of diabetes patients. Therefore, a synthesis was performed to estimate the healthcare utilization of type 2 diabetes patients using three databases: the National Panel of People with Chronic Illness or Disability (NPCD), the Netherlands Information Network of General Practice (LINH) and the National Information Service for Allied Healthcare (LiPZ). The broad healthcare utilization outline was assessed with data from the NPCG, which contains self-reported data about whether or not diabetes patients consulted various healthcare providers. Additional information about type and amount of care in primary care and diagnoses for which patients were referred to a medical specialist was derived from LINH and LiPZ. In the Dutch healthcare system, GPs act as gatekeeper for care by medical specialists. Diabetes patients need to be referred by a GP to visit a medical specialist for the first time. The diagnoses for which patients were referred to a medical specialist provide an indication about the provided care.

#### National Panel of people with Chronic Illness or Disability (NPCD)

The NIVEL research program “National Panel of people with Chronic Illness or Disability” is a nationwide panel study on the consequences of chronic illness in the Netherlands [11, 12]. Panel members are recruited from the patient files of general practices. Patients are selected according to the following criteria: a diagnosis of a non-curable chronic disease by a certified medical practitioner, an age ≥15 years, being non-institutionalized, being aware of the diagnosis, not being terminally ill [life expectancy >6 months according to their general practitioner (GP)], being mentally able to participate, and a sufficient mastery of the Dutch language. In April 2009, when the data for the current study were collected, the panel consisted of 1819 chronically ill patients recruited from 45 GP-practices in 2005, 2006, 2007 or 2008. For the purpose of this study, self-reported data on healthcare utilization in 2008 were analyzed of 316 patients diagnosed with type 2 diabetes (as index disease or comorbid condition).

#### The Netherlands Information Network of General Practice (LINH)

LINH is a representative sample of GP-practices in the Netherlands that provide routinely recorded data from their electronic medical records (EMRs). The LINH-database holds longitudinal data on morbidity, prescriptions and referrals of approximately 90 GP-practices and 350,000 listed patients [13]. The network is a dynamic pool of practices, with each year some minor changes in the number of participating practices. Diagnoses are coded using the International Classification of Primary Care (ICPC) [14].

For our analyses, we used data from practices that (a) participated in both 2007 and 2008 and (b) recorded year-round data for consultation, prescription and morbidity records in 2008. An additional inclusion criteria was set for the referral data of 2008 being recorded year round.

Patients were selected if (1) they had consulted their GP for type 2 diabetes at least once in 2007 and (2) were registered with the practice during the whole year in 2008 and (3) aged 15 or older. Type 2 diabetes patients were selected on the basis of a recorded ICPC-code T90. GPs within LINH do generally not record on ICPC-sub codes (T90.1 or T90.2), and therefore we could not distinguish between type 1 and type 2 diabetes patients on the basis of ICPC-codes. For the purpose of this study, type 1 diabetes patients were excluded on the basis of having received a prescription of insulin (ATC-code A10A), but not any oral anti-diabetic medication (ATC-code A10B) [15]. In total, data from 66 GP-practices and 9023 type 2 diabetes patients were included in the analyses. For referrals, data of 48 GP-practices and 6973 type 2 diabetes patients were included.

#### The National Information Service for Allied Healthcare (LiPZ).

LiPZ is a Dutch national representative network of about 40 physiotherapy practices, 40 practices providing exercise therapy and 30 dietetics practices [16]. Yearly, 14,000, 5000 and 3000 patients are treated in these practices, respectively. Participants routinely record healthcare related data in their EMRs, including the medical diagnoses provided by the referrer (ICPC-coded by research assistants). Type 2 diabetes patients and their number of treatment sessions cannot directly be selected from the LiPZ-data, since only the indication for the treatment (mostly musculoskeletal) is recorded and not whether there is an underlying comorbid disease. Therefore, we based our estimates of the number of treatment sessions (physiotherapist and exercise therapist) or duration of consultations (dietetics) on GPs’ referral diagnoses.

It is important to note that there is no overlap in the three databases and only aggregated data from all databases were linked. The databases of NPCD, LINH and LiPZ are registered with the Dutch data protection authority; all data are collected and handled according to the data protection healthcare standards of the authority.

### Measurements

#### Broad outline healthcare utilization

The broad outline of healthcare utilization in 2008 was assessed in the NPCD panel by means of written structured questionnaires (multiple choices). The overall response rate was 81.6%. Healthcare use was measured using the procedures of the Netherlands Health Interview Survey, in order to guarantee valid measurements [17]. Patients indicated whether or not they had used healthcare services during the previous year. We included the following disciplines/elements: GP, medical specialists (outpatient secondary care), allied healthcare, home care, hospital stay and mental healthcare.

#### Healthcare utilization in primary care

Detailed information about healthcare utilization in primary care was assessed using data from LINH and LiPZ. Healthcare utilization was categorized into contacts with GP-practice, drug prescriptions and healthcare utilization of allied healthcare.

Contacts in GP-practice were based on claims-data in the EMR in which a distinction was made between consultations (short and long), home visits (short and long) and telephone consultations with GP and primary care nurse and contacts for ‘diabetes guidance per year’. In the Netherlands, GP-practices can claim a fee for so-called ‘diabetes guidance per year’. GP-practices can claim such guidance 3–4 times a year depending on the contract they have with insurers. Within diabetes guidance GP-practices have to provide healthcare to diabetes patients based on the healthcare standard and report back about provided healthcare.

Drug prescriptions were coded automatically using the ATC classification system. A distinction was made between prescriptions for diabetes and those for other comorbid conditions, based on ATC codes at the 4 digit level.

The healthcare utilization of patients with other allied healthcare providers was based on the average number of treatment sessions (physiotherapist and exercise therapist) or duration of consultations (minutes) (dietetics) for the referral diagnoses. For example, if a diabetes patient was referred to a physiotherapist for shoulder complaints (based on LINH), the average number of treatment sessions was based on the overall median number of treatment sessions for all patients receiving physiotherapists’ care (not just diabetes patients) for shoulder complaints (based on LiPZ data). The median number of treatment sessions or minutes was determined for diagnoses for which more than 10 patients visited the allied healthcare provider. For referral diagnoses with <10 patients, the overall median was taken. In the Netherlands, patients can freely access physiotherapists and since July 2008 also exercise therapists.

#### Healthcare utilization with medical specialists

Healthcare utilization with medical specialists was obtained via NPCD database. The NPCD does not include information about the reason (diagnosis) for visiting medical specialists. In LINH referral data are available, which contain all new referrals to medical specialists including the referral diagnosis (ICPC-coded). In this study, diagnoses for which patients were referred to a medical specialist will be presented in addition to data obtained via NPCD.

#### Comorbidity

For all patients, the presence of any other chronic disease was assessed with LINH-data based on the recording of any of these diseases in 2007 or 2008. The list of chronic diseases is included in Appendix 1 and is derived from the list used by the Dutch National Institute for Public Health and the Environment [18].

#### Dutch diabetes multidisciplinary healthcare standard

In this study, health care as defined in the multidisciplinary healthcare standard of the Dutch Diabetes Federation (NDF: ‘Nederlandse Diabetes Federatie’) was compared with the healthcare utilization of type 2 diabetes patients [19]. This healthcare standard includes the care as described in the evidence based guideline of the Dutch College of GPs. Healthcare utilization was coded as ‘according to the healthcare standard’, if the provided care was mentioned in the NDF-healthcare standard. In case of contacts in general practice, patients can visit general practice for more than one complaint during a visit. In such cases, a weighted number of contacts ‘according to the healthcare standard’ was calculated (for example when both a cough and diabetes are discussed in a consultation, it is counted as 0.5 diabetes related consultation). Only healthcare utilization that could be captured with ICPC-codes was taken into account. Box 1 shows healthcare utilization for known type 2 diabetes patients based on the NDF-healthcare standard with the corresponding ICPC-codes.

### Statistical analyses

The analysis of the healthcare utilization of type 2 diabetes patients was primarily descriptive. Healthcare utilization was presented in a diagram which includes all services, and for certain parts a specific description of the healthcare utilization. Within this diagram, we highlighted the healthcare utilization that was part of the healthcare standard for diabetes to show which part of the total healthcare utilization is diabetes related and would therefore be included in a DMP for diabetes. Last, the number of other chronic diseases and most common combinations of diseases in diabetes patients was calculated based on morbidity data in LINH.

## Results

**Figure 1.**
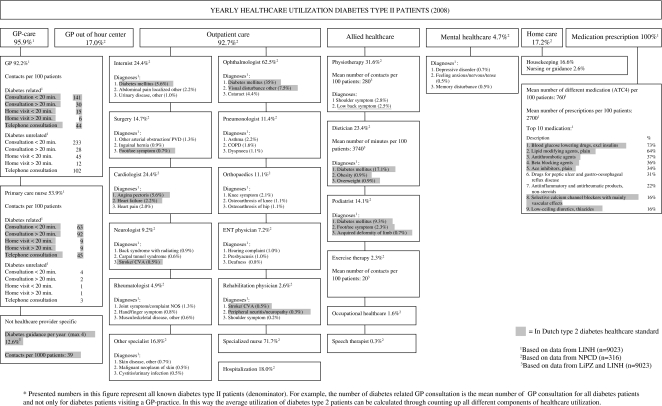
Yearly healthcare utilization of diabetes mellitus type II patients, 2008*.

Figure 1 shows an estimate of the total healthcare utilization of the type 2 diabetes patients in 2008 based on data from NPCD, LINH and LiPZ. On top of the figure for all components of healthcare (GP-care, outpatient secondary care, allied healthcare) the percentage of diabetes type 2 patients having used these care components in 2008 is shown. For example, all diabetes type 2 received drug prescriptions in 2008. Below, the components are, if possible, further divided. For example, outpatient secondary care is further divided into the different medical specialists with the percentage of diabetes type 2 patients visiting these specialists. These percentages represent the health care use of the total diabetes type 2 population, so not only diabetes related care. Under this subdivision more detailed information is shown (if available). For example for GPs, the number of diabetes related and diabetes unrelated contacts is provided and for medical specialists the referral diagnoses are shown. The percentages of patients presented here are again a percentage of the total diabetes type 2 population. In this way the average utilization of diabetes type 2 patients can be calculated through adding up all different components of healthcare utilization.

### Total healthcare utilization

Most diabetes patients (96%) used services from GP-practice, mostly provided by the GPs themselves. The total contact rate was 4.9 for diabetes related contacts and 4.3 contacts per year for non-diabetes related symptoms and diseases. Diabetes patients had on average 9.3 contacts with a GP-practice of which 53% specifically for diabetes. Most diabetes patients (93%) used also other outpatient secondary care services. Almost three quarters of the patients had consulted a specialized nurse. Frequently encountered medical specialists included ophthalmologists (63%), internists (24%) and surgeons (15%). Furthermore, 18% of the diabetes patients were hospitalized in 2008. Allied healthcare providers, including physiotherapists (32%) and dieticians (23%), were contacted by many diabetes patients. All diabetes patients received drug prescriptions in 2008 for—on average—7.6 different drugs. The most frequently prescribed drugs included blood glucose lowering drugs excluding insulin (73%) and medication for cardiovascular diseases or risk management including lipid modifying agents (64%), and antithrombotic agents (37%). The care for diabetes patients was on average provided by 4.3 (SD: 2.0) healthcare providers of different disciplines, with 20% of the patients receiving care from six or more healthcare providers. This includes healthcare providers both in primary healthcare and in secondary care, excluding specialized nurses.

### Diabetes healthcare standards

Only a part of the healthcare utilization of type 2 diabetes patient is included in the Dutch healthcare standard (highlighted in Figure 1). This included in total 36% of the contacts of GPs and 95% of the care provided by primary care nurses. Furthermore, internists’ care for diabetes, part of surgeons’, cardiologists’, neurologists’ and ophthalmologists’ care, dieticians’ care, podiatrists’ care and most of the 10 most frequently prescribed drugs was according to the standard. An important reason for the part of healthcare utilization not included in the healthcare standards is the high rate of comorbidity in diabetics: 60% of diabetes patients had another chronic disease: 31% had one, 17% two and 12% had three or more other chronic diseases. The most common comorbid conditions were coronary heart diseases (15.%), followed by dermatitis (14%) and osteoarthritis (9%) (Table 1). In Online Appendix Table A1 a full description of all other chronic diseases in type 2 diabetes patients is shown.

## Discussion

The aim of this paper was to show to what extent the total healthcare utilization of type 2 diabetes patients is disease specific and what part is not, in order to draw inferences for DMPs. Diabetes patients received care from on average 4.3 different disciplines of healthcare providers in 2008. Coordination of care is, therefore, of great importance. DMPs aim to promote the coordination of care and stimulate multidisciplinary cooperation. However, only a part of the healthcare utilization of diabetes patients is described in the healthcare standard and would therefore theoretically be included in a DMP for diabetes, as earlier shown by Hodgson and Cohen (1999) who estimated that the expenditure for diabetes care was only 40% of the total health care expenditure [20]. Diabetes related healthcare included 53% of contacts of GPs and primary care nurses, part of care of medical specialists, dieticians’ care, podiatrists’ care and most of the 10 most frequently prescribed drugs. This can partly be explained by the high rate of comorbidity in diabetes patients (60% had one or more other chronic diseases), as shown by earlier research that showed that both diabetes related and diabetes-unrelated comorbidity increases the use of medical care in diabetes patients [21]. A recent review also showed a positive association between multiple chronic conditions and health care utilization and expenditure [22]. But the fact remains that a large part of the healthcare utilization of type 2 diabetes patients would not be included in diabetes DMPs if these DMPs would focus exclusively on diabetes.

What does this mean for the coordination of care at patient level? In the case of the Netherlands, GPs function as gatekeeper and patients are registered with a GP-practice [23]. The coordination on patient level is the task of GPs. They should have an overview of all patients’ health problems and healthcare utilization. This means that healthcare is patient-oriented and GPs provide healthcare services being aware of all health conditions, which is especially important in patients with multiple chronic diseases as most diabetes patients [24]. The fact that only half of the healthcare of diabetes patients would be included in a DMP and therefore in disease oriented funding may hamper the coordination and continuity of care. Care within disease oriented funding does not have to be provided by the patient’s own GP, and therefore not all patients’ health problems and healthcare utilization could be available for and coordinated by one healthcare provider. In the Netherlands, disease oriented funding is usually contracted by groups of GPs in a specific area which not necessarily include all GPs in the region [25]. To facilitate disease management within disease oriented funding, these groups have their own specifically designed recording system. The risk of this separate recording is that health problems and utilization provided within disease oriented funding are not recorded or copied to the general EMR of the patient. Also, diabetes is one of the first diseases with disease oriented funding in the Netherlands. Disease oriented funding is also introduced for COPD and planned to be introduced for heart failure in the near future. Since 9% of the type 2 diabetes patients also suffer from COPD and 7% from heart failure, coordination and continuity of care between different DMPs will be a challenge. The introduction of DMP with disease oriented funding will therefore probably improve the quality and coordination on disease level, but could potentially violate the coordination on patient level.

The Dutch situation shows that notwithstanding the potential of DMP shown in some studies of DMPs [25], implementing DMP in countries with a strong GP system [22] could threaten the coordination on patient level which is so important in these countries. In countries with strong GP-care, policy makers must carefully design DMP, in order to prevent threats to the coordination of care on patient level. An example of a strong GP system which has integrated chronic care in the organization of primary healthcare as a whole is the UK. The UK has implemented a ‘Quality and Outcomes Framework’ (QOF) in primary care, which involves additional remuneration if GPs meet certain quality requirements for chronically ill patient groups [27]. These include indicators for quality of recording, process and outcome. In this case no threats are being made to the coordination of care on patient level. The coordination between primary and secondary care is in the UK, however, still a problem. Within countries with a less strong GP-care tradition like the US, DMPs might improve current extent of coordination on both disease and patient level, since the coordination and continuity of health care is usually not yet the responsibility of a single health care provider. In Germany, with a relatively weak primary care system, health insurers receive higher payments (separate group in risk adjustment system) for certain chronically ill patients included in certified DMPs, with the incentive for health insurers to develop DMPs [28, 29]. In this system more flexibility exists to develop DMPs for patients with multiple chronic diseases, although currently DMPs in Germany focus on single chronic diseases. This system with additional payments for patients with chronic conditions in a DMP might also be an option for countries with a strong health care system, since it does not intent to separate the healthcare for patients.

Perhaps even more importantly DMPs should not focus on a single disease, but all more common chronic conditions, since non-communicable diseases as coronary heart diseases, COPD and asthma are common in diabetes patients. By designing DMPs for the most common combinations of chronic conditions of patients, coordination of care on patient level is a better safeguarded.

### Limitations

This paper presents an overview of the healthcare utilization and comorbidity patterns of type 2 diabetes patients based on three different databases. An important limitation of this study was the construction of the healthcare utilization based on aggregated data from different databases. The current databases could not be linked at patient level, and consequently some outcomes were based on only 316 type 2 diabetes patients, whereas others have been based on more than 9000 patients. In addition, healthcare utilization as presented in this study does not reflect ideal level of healthcare, and could be different from healthcare utilization when more healthcare is delivered according to disease management. But, it does give a good insight into the potential part of care not included in national DMPs. Last, in LiPZ no information was available about diabetes as comorbidity, and therefore allied health care use could only be estimated indirectly.

## Conclusion

The care for type 2 diabetes patients involves several healthcare providers and good coordination of care is of great importance. Not all healthcare used by diabetics was diabetes related, and therefore not described in the healthcare standard, and theoretically not included in DMPs. In countries with strong GP-care this could potentially worsen the coordination and continuity of care and thereby the quality of care. To improve overall healthcare for type 2 diabetes patients focus should also be placed on the coordination between diabetes related and non-related care in designing DMPs. DMPs should be integrated in the overall organization of care.

## Figures and Tables

**Table 1.** Top-10 of other chronic diseases in type 2 diabetes patients based on LINH (n=9023), 2008

Chronic disease	Percentage of type 2 diabetes patients
Coronary heart disease	15.3%
Dermatitis	13.5%
Osteoarthritis	8.7%
Chronic obstructive pulmonary disease (COPD)	8.6%
Chronic neck or back syndrome	7.7%
Asthma	6.9%
Visual disorder	6.9%
Heart failure	6.7%
Cancer	6.7%
Transient cerebral ischaemia/cerebrovascular accident	6.3%

**Table 2.** ICPC-codes included as ‘diabetes-related’ according to the Dutch Diabetes Federation type 2 diabetes healthcare standard

F05: Visual disturbance other
F83: Retinopathy
F94: Blindness
K74: Angina pectoris
K75: Acute myocardial infarction
K76: Ischemic heart disease w/o angina
K86: Hypertension uncomplicated
K87: Hypertension complicated
K89: Transient cerebral ischemic
K90: Stroke/cerebrovascular disease,
K99.06: Peripheral diabetic angiopathy
L98: Acquired deformity limb
N94: Peripheral neuritis/neuropathy
P07: Sexual desire reduced
P08: Sexual fulfillment reduced
P17: Tobacco abuse
S06: Rash localized
S11: Other local infection skin
T02: Excessive appetite
T03: Loss of appetite
T05: Feeding problem of adult
T07: Weight gain
T08: Weight loss
T82: Obesity
T83: Overweight
T90: Diabetes mellitus
T93: Lipid disorder
X24: Fear of sexual dysfunction female
Y07: Impotence NOS
Y24: Fear of dysfunction male

**Appendix 1:** Table A1. List of chronic diseases with ICPC-code and percentage of type 2 diabetes patients with the chronic disease

Chronic disease	ICPC-code	Percentage of type 2 diabetes patients
Tuberculosis	A70	0.0%
HIV-infection/AIDS	B90	0.0%
Cancer	A79, B72, B73, D74, D75, D77, L71, N74, R84, R85, S77, T71, U75, U76, U77, W72, X75, X76, X77, Y77, Y78	6.7%
Peptic or duodenal ulcer	D85, D86	0.9%
Chronic enteritis/ulcerative colitis	D94	0.5%
Visual disorder	F83, F84, F92, F93, F94	6.9%
Hearing disorder	H84, H85	1.5%
Congenital anomaly cardiovascular	K73	0.0%
Coronary heart diseases	K74, K75, K76	15.3%
Heart failure	K77	6.7%
Transient cerebral ischaemia/ cerebrovascular accident	K89, K90	6.3%
Chronic neck or back syndrome	L83, L84, L85, L86	7.7%
Rheumatoid arthritis	L88	1.8%
Osteoarthritis	L89, L90, L91	8.7%
Osteoporosis	L95	3.2%
Congenital anomaly neurological	N85	0.0%
Multiple sclerosis	N86	0.1%
Parkinson	N87	0.6%
Epilepsy	N88	0.8%
Chronic alcohol abuse	P15	0.9%
Dementia	P70	1.6%
Schizophrenia	P72	0.3%
Anxiety disorder, other neurosis	P74, P79	2.0%
Depression	P76	5.8%
Mental retardation	P85	0.1%
Chronic obstructive pulmonary diseases (COPD)	R91, R95	8.6%
Asthma	R96	6.9%
Dermatitis	S87, S88	13.5%
Anorexia	T06	0.0%

**Box 1.** Health care utilization for known type 2 diabetes patients based on Dutch Diabetes Federation type 2 diabetes healthcare standard

***Check-ups by GP and primary care nurse***
3-montly check-up: wellbeing, hypo- or hyperglycemia, nutritional problems or exercise advice and medication, body weight, fasting blood glucose levels, blood pressure (if patient uses antihypertensive drugs), foot examination (if patient had ulcus, acquired deformity of limb or serious neuropathy foot)
Yearly check-up: possible visual problems, cardiovascular diseases, neuropathy or sexual problems, lifestyle aspects as smoking status, exercise and alcohol use, blood pressure, body weight, foot examination, inspection insuline injection sites (if patients uses insuline), eye fundus examination. Laboratory measures: fasting blood glucose, HbA1c, creatinine levels, potassium levels (if patient uses diuretic or RAS inhibitor), creatinine clearance, albumin creatinine-ratio or albumin urine levels (if patients has a life expectancy of minimal 10 years), fasting lipids spectrum
***Medication***
Diabetes: oral blood glucose lowering drugs, insulin
Risk factor cardiovascular diseases: lipid modifying agents (recommended for almost all type 2 diabetes patients), diuretics, ACE inhibitors, angiotensin-ii-antagonists, beta blocking agents, calcium channel blockers, antithrombotic agents
Superficial foot ulcer: oral antibiotic
***Consultation other healthcare providers***
Internist (including nephrologistadjustment insulin (when knowledge not available in GP-practice), insufficient correction postprandial blood glucose levels with two-times daily insulin, diabetes ulcer, low creatinine clearance, serious hyperglycemia or hyperglycemic coma, pregnant women or women with pregnancy wish
Dietician: for extensive nutrition advice
Ophthalmologist: retinaphotography (if not available in GP-practice), assessment of retinaphotography (if expertise not available in GP-practice), deviations eye fundus
Podotherapist: callous and/or pressure sites without signs of peripheral vascular disease
Surgeon: diabetes ulcer
Orthopedic: diabetes ulcer
Dermatologist: diabetes ulcer
Based on the described healthcare utilization the following ICPC-codes were coded as ‘according to the healthcare standard’ see Table 2.
